# Reproductive behaviour of the ocean triggerfish *Canthidermis sufflamen* (Tetraodontiformes: Balistidae) in the shallow waters of the marine‐protected area of El Hierro Island (Canary Islands)

**DOI:** 10.1111/jfb.70374

**Published:** 2026-02-26

**Authors:** Alberto Rodríguez‐Díaz, Raül Triay‐Portella, José A. González, José G. Pajuelo

**Affiliations:** ^1^ Grupo en Biodiversidad y Conservación (BIOCON), IU‐ECOAQUA, Universidad de Las Palmas de Gran Canaria Las Palmas de Gran Canaria Canary Islands Spain; ^2^ Applied Marine Ecology and Fisheries Division (EMAP), University Research Institute for Environmental Studies and Natural Resources (i‐UNAT), University of Las Palmas de Gran Canaria Las Palmas Spain

**Keywords:** mating system, ocean triggerfish, sex ratio, size at maturity, spawning season

## Abstract

The abundance of *Canthidermis sufflamen* has increased worldwide, including around the Canary Islands, due to global ocean warming. The reproductive behaviour of this species was studied within a marine‐protected area (MPA) and its adjacent areas off the island of El Hierro (Canary Islands). This oceanic species starts to move from pelagic habitats to inshore waters at the end of April. Spawning takes place asynchronously from June to September, with peak activity in July–August. Males establish territories and build several benthic nests on sandy bottom for multiple females. Each male covers an area ranging from ~21 to 135 m^2^. The maximum number of territorial males was observed in July–August, whereas the minimum was recorded in May. A polygynous mating system was observed, with a sex ratio skewed towards females during the reproductive period. In each male territory within the MPA, one to five females were observed, whereas only one to three females were recorded outside the MPA. During the spawning season, males exhibit a darker body colouration, indicative of sexual dichromatism. Reproductive activity ends in September, although some males and females remain until October, providing biparental care. Females care for benthic eggs, staying within ~2 m of the nest. By the end of October, all individuals leave inshore waters and return to the ocean pelagic habitats. No specimens are present in coastal waters from November to March. The MPA plays a key role in ensuring successful reproduction of this species, acting as a source of colonisation for the surrounding regions and neighbouring islands.

## 1 INTRODUCTION

Triggerfishes (Balistidae) are distributed throughout the world's oceans, occurring in tropical to temperate seas (Matsuura, 2015). In the Canary Islands, six species of Balistidae family have been recorded in the past three decades (Brito et al., 1995, 2002): *Balistes capriscus* Gmelin, 1789; *Balistes punctatus* Gmelin, 1789; *Balistes vetula* Linnaeus, 1758; *Canthidermis maculata* (Bloch, 1786); *Canthidermis sufflamen* (Mitchill, 1815); and *Melichthys niger* (Bloch, 1786).

The distribution ranges of these species have expanded worldwide because of global ocean warming (Brito et al., 1995; González et al., 2020; Tejera, 2018, 2022). In the Canary Islands, the occurrence of the ocean triggerfish (*C. sufflamen*) has increased significantly, progressing from isolated records ~30 years ago to the establishment of a targeted artisanal fishery for more than two decades (González et al., 2020). The first sporadic records of landings reported by the government of the Canary Islands date back to 1998, with catches exceeding 8 t 10 years later. Currently, annual landings slightly exceed 11 t on the island of El Hierro, representing approximately 20% of total shallow demersal catches and 8% of their economic value. Despite its growing ecological and fisheries relevance, information on the biological characteristics of this species within its natural distribution range remains scarce (Triay‐Portella et al., 2015).

The ocean triggerfish is distributed in the western Atlantic (from Massachusetts through the Gulf of Mexico to the Lesser Antilles and Bermuda) (Matsuura, 2015), the central Atlantic (St Paul's Rocks, Ascension Island, and St Helena) (Feitoza et al., 2003) and the eastern Atlantic (São Tomé and Príncipe, and the archipelagos of the Azores, Madeira, the Canary Islands and Cape Verde) (Afonso et al., 1999; Brito et al., 1995; Wirtz et al., 2008). It is considered a warm‐water fish species occurring in tropical and subtropical waters (Brito et al., 1995), and it is typically observed near the surface below floating objects, in oceanic pelagic and inshore habitats (Gasparini & Floeter, 2001), as well as on offshore reefs and drop‐offs near deep waters (Feitoza et al., 2003; Heyman & Kjerfve, 2008). The species is commonly found at depths between 5 and 60 m (Feitoza et al., 2003; Gasparini & Floeter, 2001; Heyman & Kjerfve, 2008), although it has also been recorded in very shallow waters (1–2 m) (Kuhlmann et al., 2011). The ocean triggerfish is a carnivorous species that feeds mainly on zooplankton and benthic invertebrates (Gasparini & Floeter, 2001).

The reproductive ecology of many Balistidae species shares several common features (Kawase, 2002). Species in this family are dioecious (gonochoristic). Males establish territories and construct benthic nests on sandy bottoms or sandy reef substrates to attract females for spawning. Prior to fertilisation, females move among male territories, visiting and inspecting nests (Blumer, 1982; Kawase, 2003; Simmons & Szedlmayer, 2012). In many species, spawning occurs in pairs at sunrise. During mating, individuals of both sexes touch each other's abdomen, releasing adhesive benthic eggs (Gladstone, 1994; Kawase, 2002). After fertilisation, either maternal or biparental egg care may occur (Kawase, 2002). Males defend their territories surrounding the nests, whereas each female cares for its nest by driving away fish of other species (Fricke, 1980; Kawase, 2002; Simmons & Szedlmayer, 2012).

Despite the importance of this species as a target of global fisheries (Alarcón et al., 2017; González et al., 2020; Kelly‐Stormer et al., 2017) and as a keystone component of benthic communities (Clemente et al., 2010; McClanahan, 1995, 1999), knowledge of its reproductive behaviour remains limited. This study evaluates whether the reproductive patterns and behaviours of the ocean triggerfish align with those described for other members of the family Balistidae. Therefore, the main objective is to characterise the reproductive behaviour of the ocean triggerfish and to compare it with established reproductive traits reported for other balistid species. Although the Canary Islands constitute a marginal distribution zone, the species' ability to persist and reproduce there implies that reproductive parameters may deviate from those in its natural range. If these parameters remain consistent, it would indicate a high potential for the establishment of populations further north. The study was conducted within a marine‐protected area (MPA) and its adjacent areas around the island of El Hierro to minimise potential disturbance caused by fishing activities. The biological information obtained here provides a baseline for future comparative studies in other regions of the species' distribution and may contribute to the management and conservation of the ocean triggerfish within the Canary Island fishery.

## 2 MATERIALS AND METHODS

**FIGURE 1:**
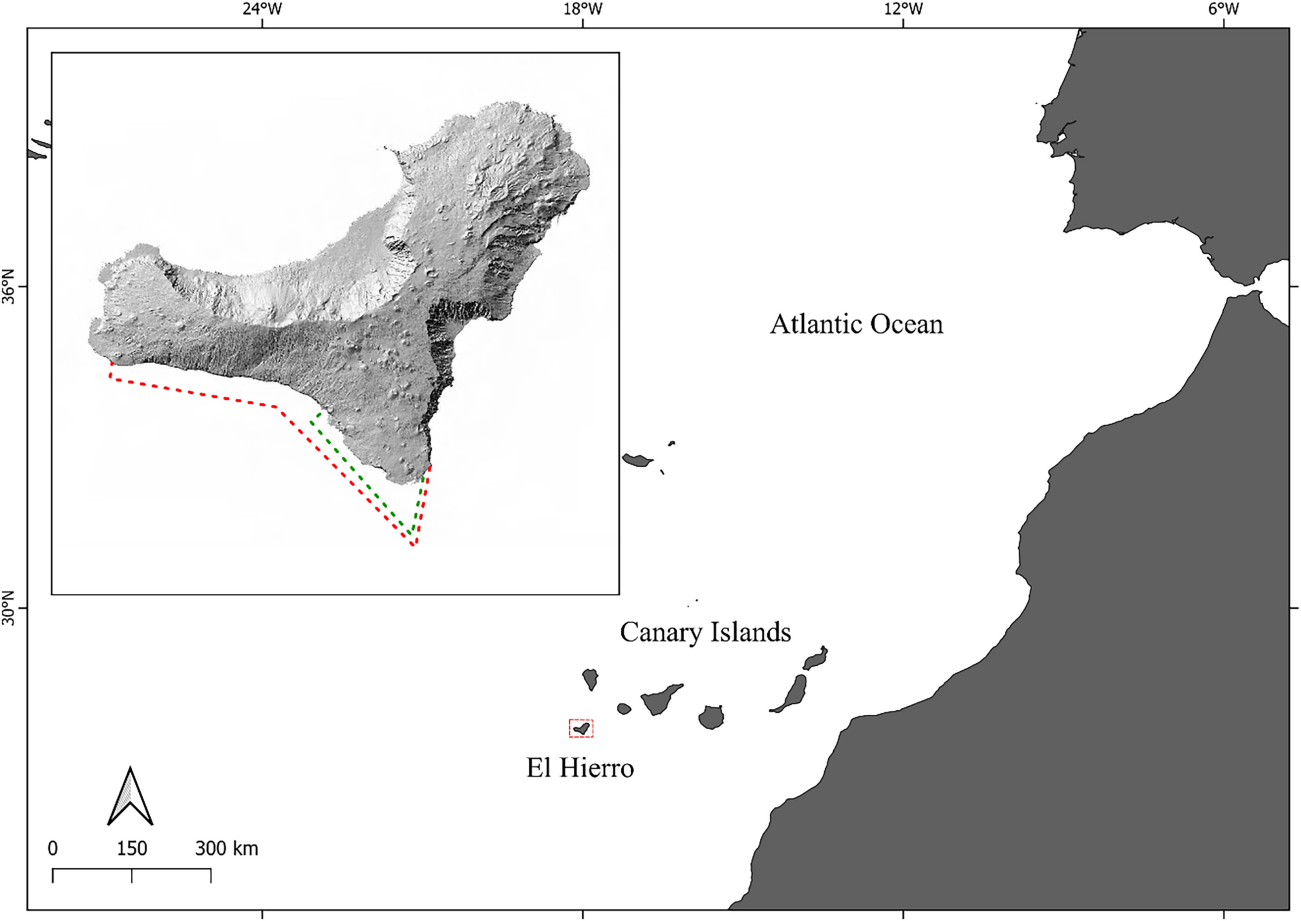
Sampling location off El Hierro Island, Canary Islands. The green line indicates the boundaries of the marine‐protected area, whereas the red line indicates the area of the El Mar de Las Calmas.

Specimens of ocean triggerfish were observed within the La Restinga‐Mar de Las Calmas Marine Protected Area (MPA), on the island of El Hierro (Canary Islands), as well as in adjacent areas of El Mar de Las Calmas (Figure 1). The MPA was established in 1996 and covers an area of 7.46 km^2^ (BOE, 1996). It is located in the south‐western part of El Hierro, the westernmost island of the Canary archipelago. Unlike other Canary Islands that are more strongly influenced by the African coastal upwelling, this region is characterised by more tropical water conditions. The upwelling generates a temperature gradient that increases from the African coast towards El Hierro, with a difference of ~7°C.

This study was conducted throughout 2021. Only from April to October 2021 did scuba divers and a Deep Trekker DTG2 remotely operated vehicle (ROV) record the presence and reproductive behaviour of the ocean triggerfish at depths of 5–20 m. The maximum observation time was constrained by scuba diving limits and the operational autonomy of the ROV, lasting between 1.0 and 1.25 h depending on current conditions 2 days per month. During the remaining months of the year, the species was not detected either in coastal areas or in artisanal fishery catches.

Sampling was conducted both inside and outside the MPA, covering areas of 1000 m^2^ in each zone. ROV laser scaling and distance measurements were used to estimate spatial coverage and distances. The ROV was also used to record male movements. Behavioural variables recorded included the number of females per male territory, distances between nests, territory area, aggressive territorial behaviour, maternal or biparental care, substrate type and number of nests. Kruskal–Wallis and χ^2^ tests were applied to assess differences between areas. From November to March, ocean triggerfish individuals were absent from inshore waters (<100 m depth).

During the same period, 397 ocean triggerfish specimens captured by artisanal fisheries were analysed in the laboratory to assess their reproductive status. Specimens were measured for fork length (FL) to the nearest millimetre and weighed for total weight (TW), eviscerate weight and gonadal weight to the nearest gram, gram and 0.01 g, respectively. A Mann–Whitney test was used to evaluate significant differences in FL and TW between sexes. Additionally, a Kolmogorov–Smirnov (K–S) test was applied to compare the differences in the size‐frequency distributions between sexes.

Gonads were fixed in PanReac formaldehyde (3.7%–4.0% w/v), buffered to pH 7 and stabilised with methanol for histological analysis. The onset of sexual maturity was determined through microscopic examination of gonadal tissue (Brown‐Peterson et al., 2011). Gonadal samples (*n* = 397) were dehydrated through a graded ethanol series, cleared in isoparaffin and embedded in Paraplast paraffin. Sections (5 μm thick) were stained with Harris haematoxylin and counterstained with eosin.

The terminology proposed by Brown‐Peterson et al. (2011) and Lowerre‐Barbieri et al. (2011) was used to describe oocyte development, including primary growth (PG) oocytes, cortical alveolar (CA) oocytes, primary vitellogenic (Vtg1) oocytes, secondary vitellogenic (Vtg2) oocytes, tertiary vitellogenic (Vtg3) oocytes, oocyte maturation (OM) and the substage of OM: germinal vesicle migration (GVM), yolk coalescence and germinal vesicle breakdown (GVBD). The criteria of Brown‐Peterson et al. (2011) and Lowerre‐Barbieri et al. (2011) were also applied to identify postovulatory follicle (POF) complexes and atretic (A) oocytes. Spermatogenesis was classified into four histological stages, modified from Brown‐Peterson et al. (2011): spermatogonia (Sg), spermatocytes (Sc), spermatids and spermatozoa.

The spawning season was delimited using reproductive phases. The onset of the spawning season was defined by the appearance of females in the spawning‐capable phase, whereas the end of the spawning season was indicated by the presence of numerous females in the regressing phase (Brown‐Peterson et al., 2011). Size at maturity (*L*_50_) was estimated as the length at which 50% of males or females were mature during the study period (Saila et al., 1988).

A gonado‐somatic index (GSI) was used to examine changes in gonadal development throughout the spawning season (Anderson & Gutreuter, 1983). Monthly mean GSI values were compared using a Kruskal–Wallis test to assess temporal variation.

## 3 RESULTS

**FIGURE 2:**
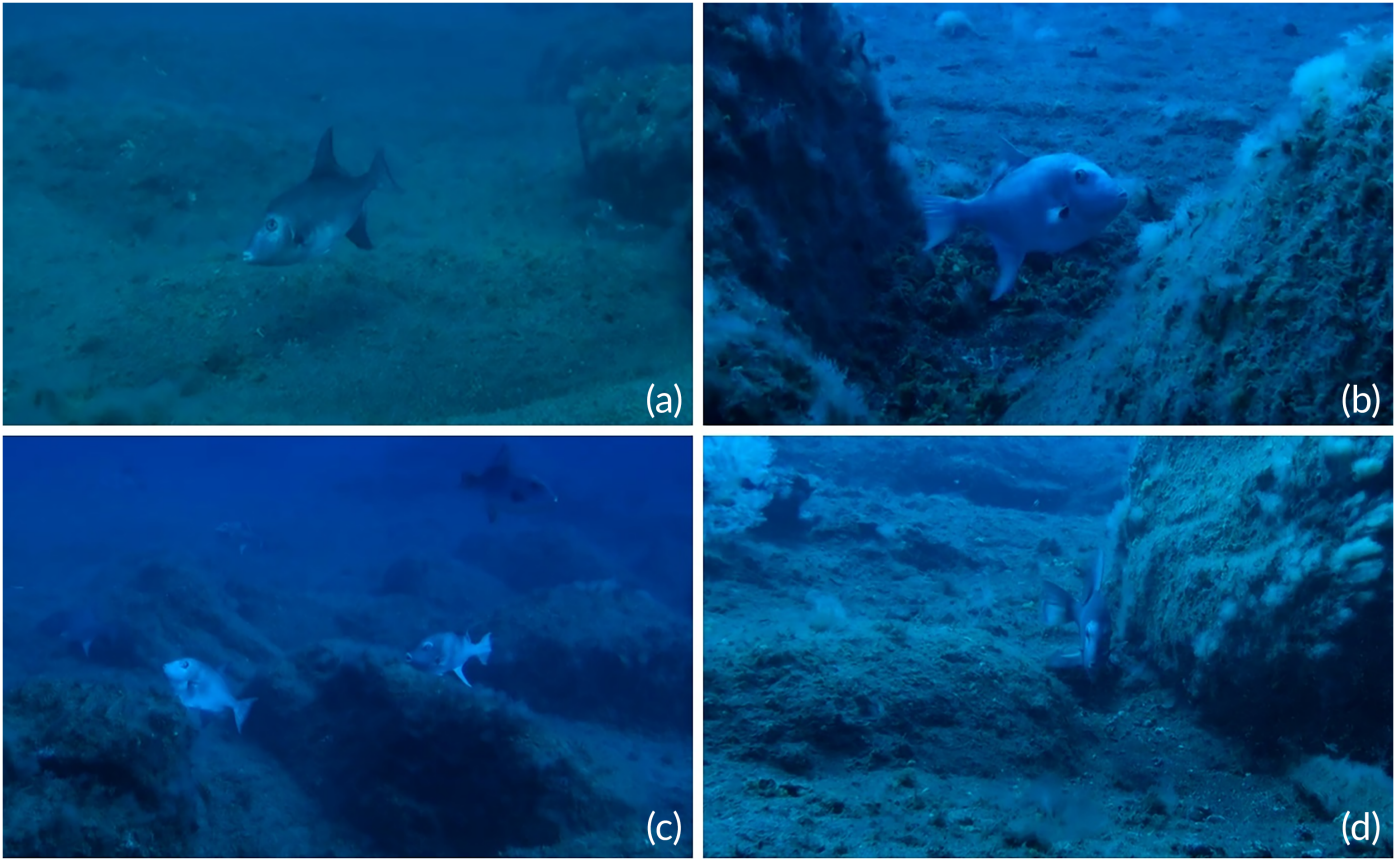
Specimens of ocean triggerfish *Canthidermis sufflamen* during spawning season in El Hierro (Canary Islands). (a) Male with a dark body colouration. (b) Female with a light colouration. (c) One male and two females in the same territory. (d) Female guarding a nest with eggs.

Males exhibited territorial and aggressive behaviour, surrounding several nests and exhibiting a dark body colouration (Figure 2a,b). Each male covers an approximate surface area ranging from 21 to 135 m^2^ (mean *x¯*=56.83 ± 32.4 m^2^), with significantly larger areas inside than outside the MPA (*H* = 21.1, *p* < 0.001). The number of male territories containing females differed significantly between areas (*Χ* ^2^ = 12.25, *p* < 0.001).

The number of territorial males changed throughout the months. The maximum was observed in July and August (*n* = 12), whereas the minimum was observed in May (*n* = 2). The number of females per male territory also differed significantly between areas (*H =* 10.4, *p <* 0.001). Inside the MPA, between one and five females were observed per male territory (*x¯*=2.67 ± 3.2), with females remaining close to the nests. These nests were located on sandy bottoms and were spaced ~7–10 m apart (Figure 2c,d). Outside the MPA, only one to three females were observed per male territory ($\bar{x}=1.5\pm 0.78$).

The number of male territories recorded ranged from five in July and August to one in May. No nests were observed in April. Females deposited their eggs in the sand within the nests, where fertilisation by the male occurred. After spawning, females remained near the nest for 23 and 29 days. No significant differences were observed between areas in the duration of female nest attendance after egg deposition (*H* = 0.68, *p* = 0.41). Males remained within the territory, whereas females were present near the nests.

**FIGURE 3:**
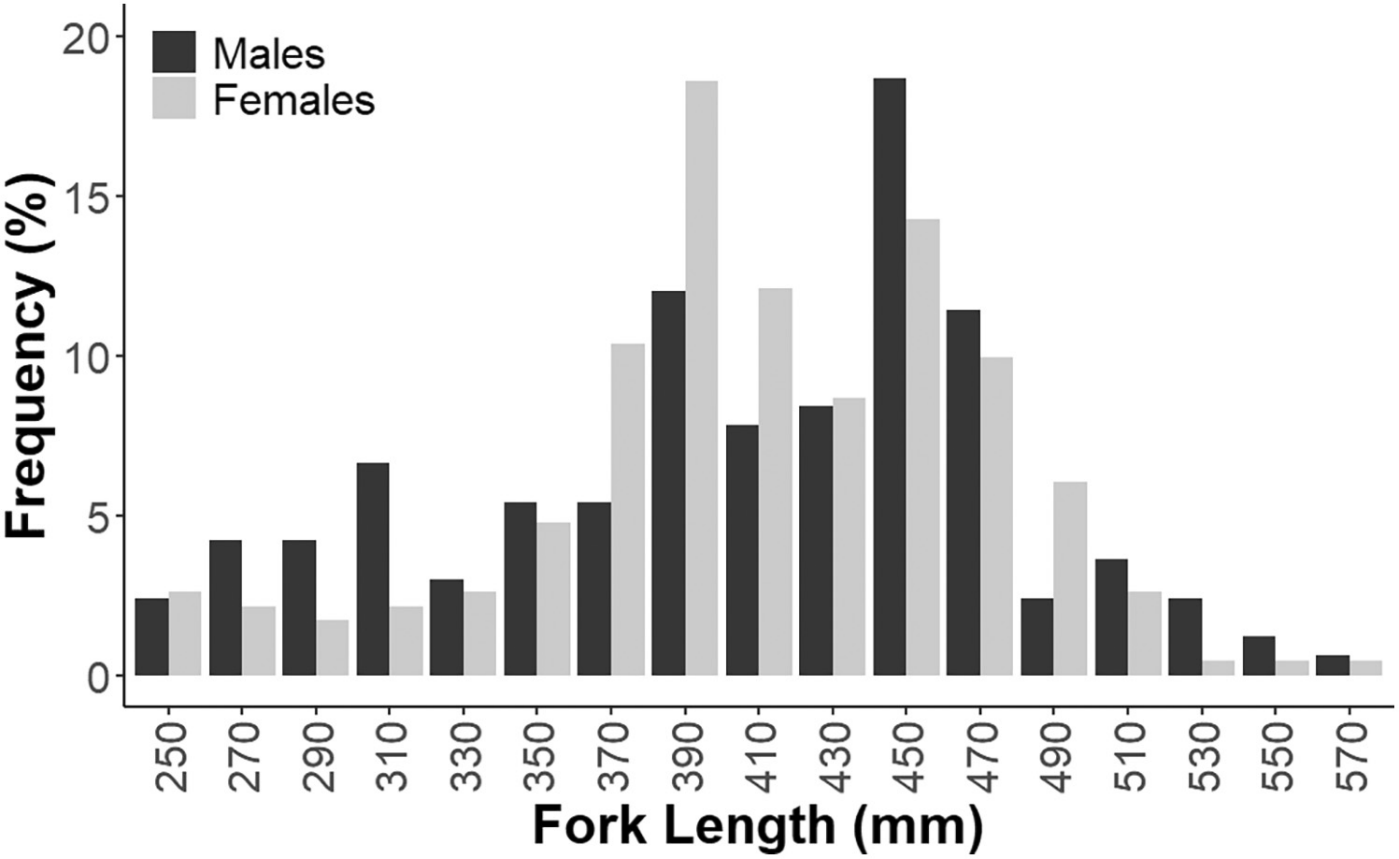
Size‐frequency distribution (fork length, mm) for each sex of the ocean triggerfish *Canthidermis sufflamen* of El Hierro Island (Canary Islands).

A total of 397 ocean triggerfish were collected and analysed, of which 166 were males (42%) and 231 were females (58%). The sex ratio was significantly skewed in favour of females (*Χ* ^*2*^ = 10.64, *p* < 0.001). The smallest fish recorded was a female (251 mm FL, 487 g TW), and the largest individual was also a female (571 mm FL, 3854 g TW) (Figure 3). No significant differences were found between sexes in size (*U* = 19.082, *p* = 0.936) or weight (*U* = 18.807, *p* = 0.746). Males and females exhibited similar size‐frequency distributions (*Z* = 0.006, *p* = 0.936) (Figure 3). Paired K–S tests revealed no significant temporal changes in the size‐frequency distributions of either sex from May to October (*Z* ≤ 3.256, *p* ≥ 0.071).

**FIGURE 4:**
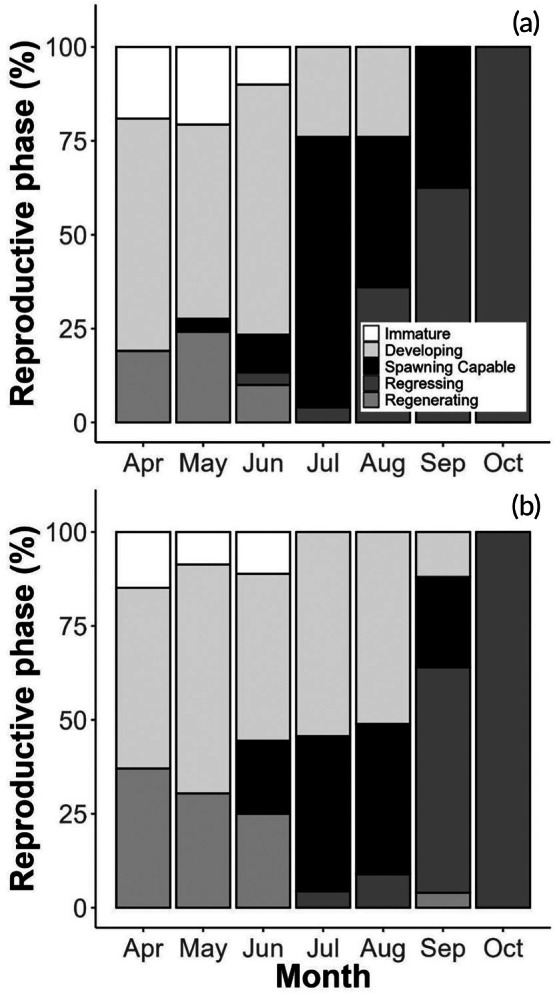
Percentage of occurrence for each reproductive phase of (a) males and (b) females of the ocean triggerfish *Canthidermis sufflamen* of El Hierro (Canary Islands).

**FIGURE 5:**
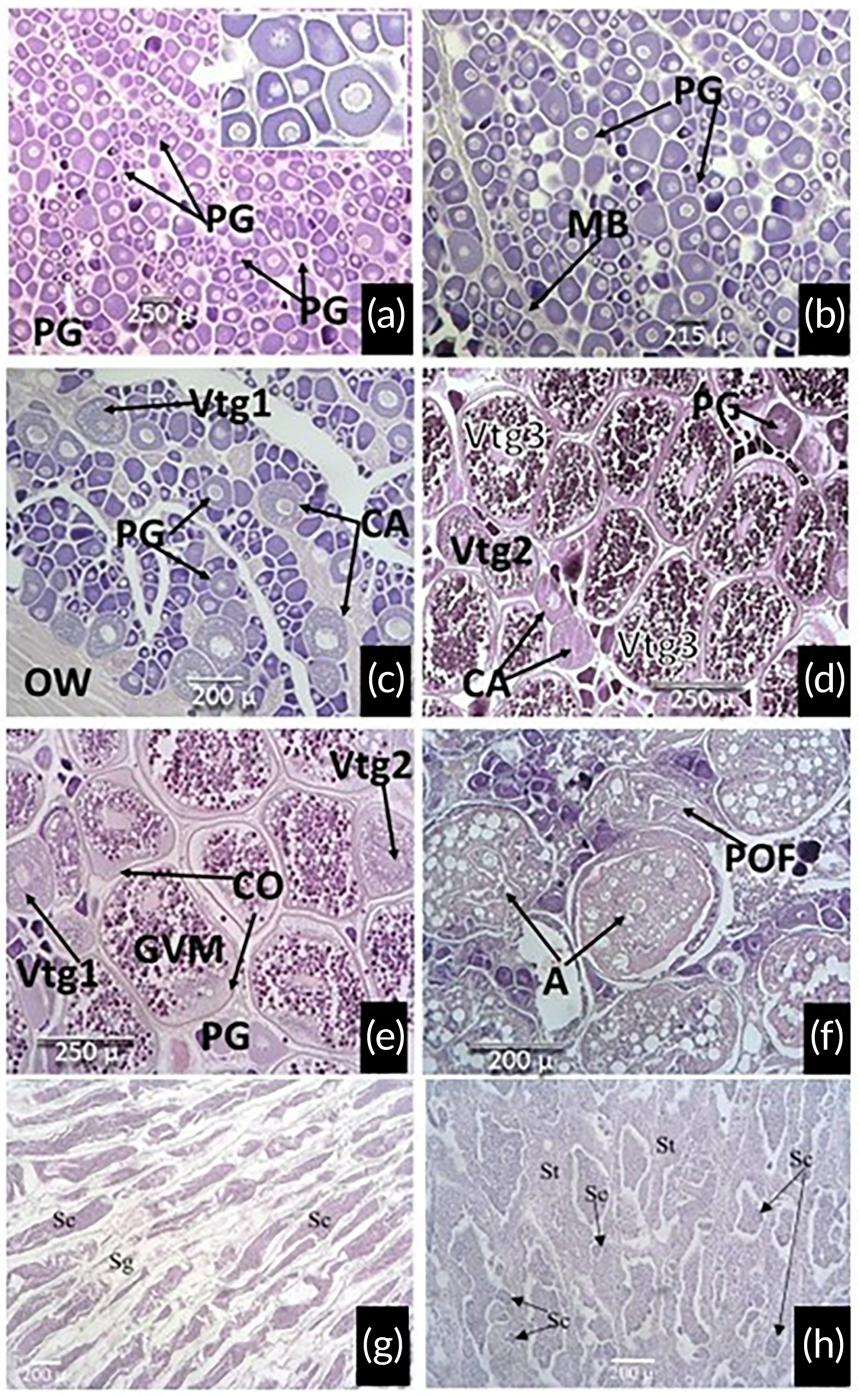
Microimages of the histological sections of the ovary and the testis of the ocean triggerfish *Canthidermis sufflamen*. (a) Immature phase [primary growth (PG) oocyte]. Details of the different shapes and sizes of PG oocytes, some with nucleoli flattened against the nuclear membrane and others with nucleoli free in the nucleoplasm. (b) Regenerating phase [muscle bundle (MB)]. (c) Developing phase [cortical alveolar (CA) oocyte, ovarian wall (OW), primary vitellogenic (Vtg1) oocyte]. (d) Spawning‐capable phase [secondary vitellogenic (Vtg2) oocyte, tertiary vitellogenic (Vtg3) oocyte, presence of recent postovulatory follicles]. (e) Actively spawning subphase [germinal vesicle breakdown (GVBD), germinal vesicle migration (GVM), yolk coalescence (CO)]. (f) Regressing phase [atresia (A), postovulatory follicle (POF) complexes]. (g) Immature phase [spermatogonia (Sg), spermatocytes (Sc)]. (h) Regenerating phase [Sc, spermatids (St)].

The monthly progression of gonadal maturity stages in males and females is shown in Figure 4. In males, the immature phase was observed mainly in April, May and June (Figure 4a). The developing phase occurred from April to July. The spawning‐capable phase extended from May to September, whereas the regenerating phase was recorded in April, May and June. The regressing phase was most prevalent in August, September and October (Figure 4a). Males in the immature phase exhibited Sg and Sc, whereas the spawning‐capable phase was characterised by abundant Sc (Figure 5g,h).

In females, the immature phase occurred in April, May and June (Figure 4b) and was identified by the presence of PG oocytes, the absence of muscle bundles (MB) and a thin ovarian wall (OW) (Figure 5a). The developing phase was present in all months, except October (Figure 4), and was characterised by the presence of PG, CA, Vtg1 and Vtg2 oocytes (Figure 5c). The spawning‐capable phase occurred from June to September (Figure 4b) and was characterised by the appearance of Vtg3 oocytes and POFs (Figure 5d). The actively spawning subphase was observed mainly in July and August and was distinguished by GVM (Figure 5e).

**FIGURE 6:**
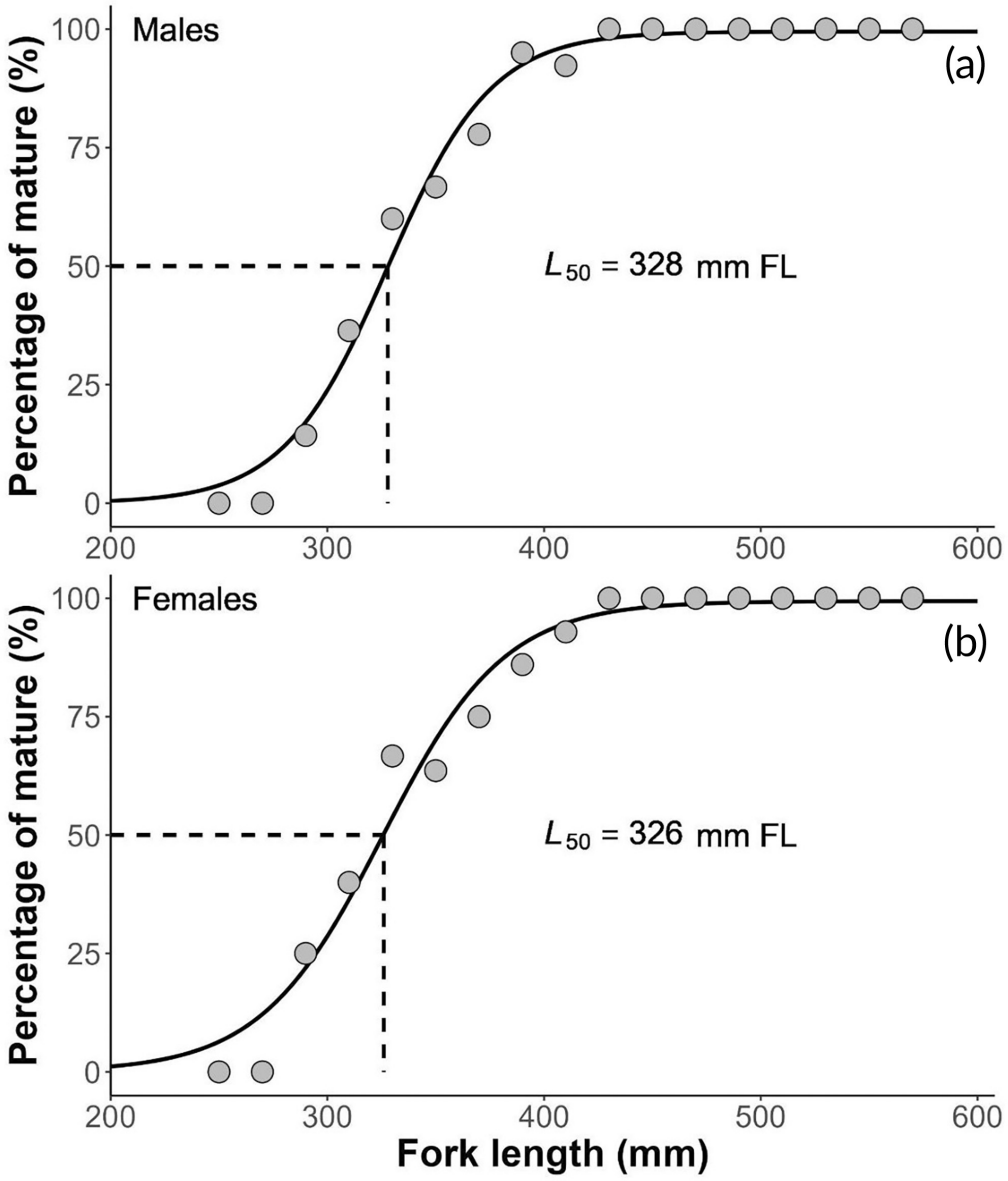
Size at first maturity (*L*_50_) of (a) males and (b) females of the ocean triggerfish *Canthidermis sufflamen* of El Hierro (Canary Islands).

The regressing phase began in July and extended until October (Figure 4b) and was characterised by the presence of atretic oocytes, a small number of vitellogenic oocytes and POFs (Figure 5f). The regenerating phase was observed mainly between April and June (Figure 4b) and was recognised by the presence of PG oocytes, MB and a thick OW (Figure 5b). Size at maturity (*L*_50_) was estimated at 328 mm FL for males and 326 mm FL for females (Figure 6).

**FIGURE 7:**
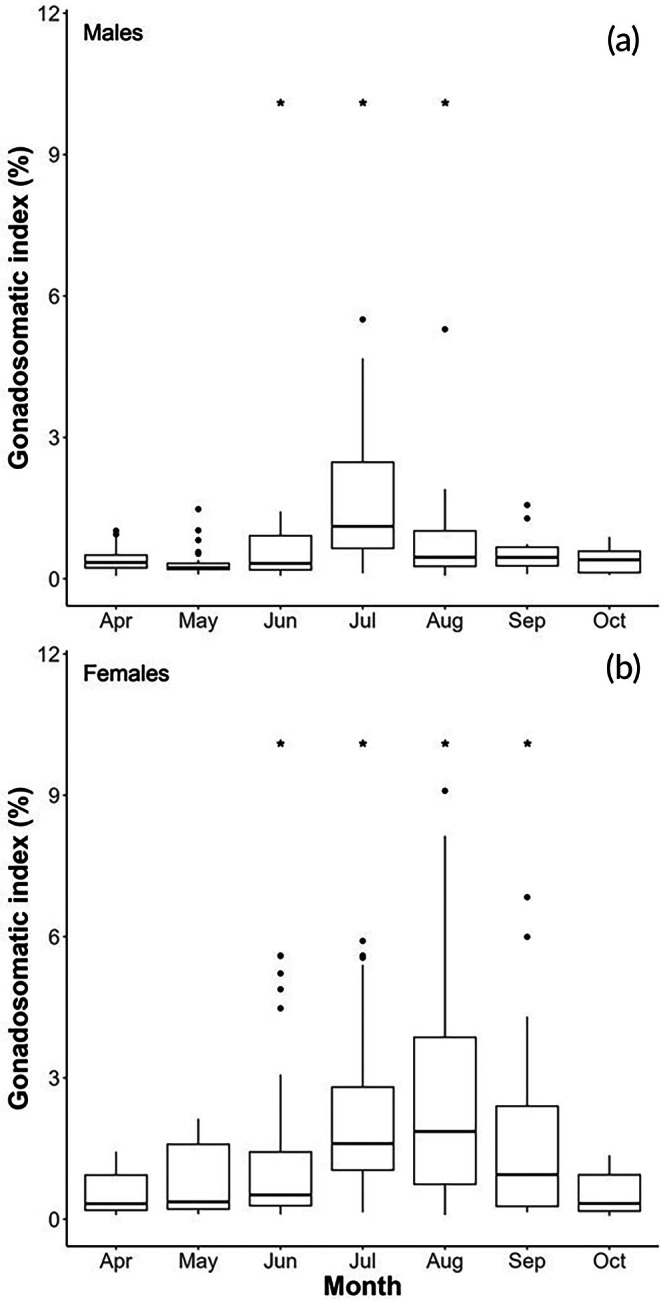
Gonado‐somatic index (GSI) of (a) males and (b) females of the ocean triggerfish *Canthidermis sufflamen* of El Hierro (Canary Islands). Significant differences between months are indicated by asterisks (*p* < 0.05).

The GSI of males exhibited the highest values in July and August, whereas that of females exhibited peak values in July, August and September (Figure 7). Mean GSI values varied significantly among months for both males (*H* = 24.325, *p* < 0.001) and females (*H* = 54.366, *p* < 0.001).

## 4 DISCUSSION

The reproductive behaviour of the ocean triggerfish observed off El Hierro Island is consistent with patterns reported for other Balistidae species. These findings provide valuable insights for the sustainable management of fisheries targeting this species, both within the study area and potentially in other regions.

The Canary Islands are characterised by oligotrophic waters (Schmoker et al., 2012), which may limit growth rates and delay sexual maturation. Furthermore, the ocean triggerfish was first reported in the Canary Islands only 28 years ago (Brito et al., 1995), and its presence has been associated with a broader tropicalisation process affecting the region (Brito et al., 2002). As the Canary Islands lie near the northern limit of the species' distribution, environmental conditions may not be optimal for growth and reproduction. However, ongoing global warming could modify reproductive traits in the future as seawater temperatures and ecological tolerance approach the species' thermal optimum. Despite these constraints, the maximum sizes observed in the Canary Islands during the study are comparable to those reported from other regions (Alarcón et al., 2017; Froese & Pauly, 2025).

Specimens <250 mm FL were neither captured by the artisanal fishery nor observed during scuba diving or ROV surveys. Therefore, the individuals observed and collected in this study represent only those that migrated from oceanic habitats to coastal waters for reproduction. This pattern suggests that juveniles may remain in pelagic or oceanic environments and move to inshore habitats only upon reaching sexual maturity. Consequently, the minimum size recorded in this study may be a reliable indicator of the onset of maturation. However, smaller specimens have been reported in coastal waters in other regions (Alarcón et al., 2017), which may reflect the differences in local environmental conditions.

The lack of differences in mean size and weight, as well as in size‐frequency distribution, indicates that the ocean triggerfish is not a sexually size dimorphic species. In other Balistidae species (e.g. *B. capriscus*, *B. vetula*), adult males are larger than females (Kelly‐Stormer et al., 2017; Rivera Hernández et al., 2019). For ocean triggerfish in Venezuela, Alarcón et al. (2017) also found no evidence of sexual dimorphism in size or weight. The absence of size dimorphism likely reflects the ecological demands of an ocean pelagic habitat, in which larger body size enhances survival against predators and increases foraging success (Brooks & Dodson, 1965). This constraint appears less stringent in demersal Balistidae species such as *B. capriscus* present in the study area.

Throughout the study, the sex ratio was skewed in favour of females. This pattern is attributed to the polygynous mating system observed in the ocean triggerfish, in which several females occupy a single male's territory. Polygyny has been widely reported in Balistidae species, including *B. capriscus*, *Odonus niger* (Rüppell, 1836); *Pseudobalistes fuscus* (Bloch & Schneider, 1801); *Sufflamen chrysopterum* (Bloch & Schneider, 1801); *Sufflamen verres* (Gilbert & Starks, 1904); and *Rhinecanthus aculeatus* (Linnaeus, 1758) (Kawase, 2003; Simmons & Szedlmayer, 2012; Takamoto et al., 2003).

Based on underwater observations and the results obtained, the ocean triggerfish is a gonochoristic species that reproduces via external fertilisation. Individuals begin migrating to inshore waters at the end of April, with peak spawning activity occurring during July, August and September. Thus, the spawning season concludes in September, and in October individuals leave inshore waters and return to ocean pelagic habitat after a period of biparental care. From November to March, no individuals were observed in inshore waters. The spawning season can be identified by the presence of individuals in coastal habitats. This seasonality pattern may be driven by a combination of several environmental factors, including temperature, photoperiod and nutrient availability (Tejera, 2018).

The reproductive behaviour observed in ocean triggerfish males and females closely matches that described for other Balistidae species [e.g. *B. capriscus*, *B. vetula*, *Pseudobaliste flavimarginatus* (Rüppell, 1829)], in which males establish territories and build benthic nests (Gladstone, 1994; Kawase, 2002; Simmons & Szedlmayer, 2012). Furthermore, males exhibited dark body colouration (i.e. sexual dichromatism) during the spawning season. Sexual dichromatism in fishes often involves conspicuous male colouration that functions in female attraction during breeding (Berglund et al., 1996). Such contrasting colours act as visual signals of individual condition and reproductive ability, and also play a role in establishing social hierarchies, territory defence, courtship and spawning readiness. Dominant males typically exhibit more intense colour patterns (Badyaev & Hill, 2000; Berglund et al., 1996; Salgado Cruz et al., 2025). Sexual dichromatism has been reported previously for the ocean triggerfish in Belize (Heyman & Kjerfve, 2008) and in several other Balistidae species (Kawase, 2003; Simmons & Szedlmayer, 2012; Takamoto et al., 2003).

The brief embryonic development period, together with asynchronous oocyte development (characterised by multiple stages of maturity) and the presence of POFs in spawning‐capable females, indicates that protracted nest care involves the release of multiple spawning batches (or pulses) within the same nest (Mouchlianitis et al., 2025; Tejera, 2022). In this study, variability in female GSI values was greater than that observed in males, indicating that GSI is a better indicator of spawning‐season duration in females. In April and May, female GSI values were low (<1.2%), coinciding with the lowest number of nests observed. During these months, females exhibited Vtg2 oocytes as the most advanced stage. In contrast, during June, females presented Vtg3 oocytes, which accumulated more lipids and are larger than Vtg2 oocytes (Brown‐Peterson et al., 2011). This explains the elevated GSI values recorded in June, July, August and September, corresponding to the highest nesting activity. After spawning, GSI values declined, coinciding with a period dominated by biparental care.

The eggs are benthic, a trait shared with many other Balistidae species (Kawase, 2002; Rivera Hernández et al., 2019; Simmons & Szedlmayer, 2012). Females provide maternal care by remaining on or close to the nests (within 2 m), whereas males guard active nests within their territories. This pattern of parental care has been widely documented in Balistidae species (Fricke, 1980; Kawase, 2002; Simmons & Szedlmayer, 2012), suggesting that it represents a family‐level reproductive strategy.

Temperature and its ongoing increase play a major role in driving range contractions or expansions through changes in habitat suitability relative to species' thermal optimum abiotic conditions (Baudron et al., 2020; Kutsyn, 2025). The ocean triggerfish can therefore be considered a mobile species whose distribution is closely linked to global warming. Its northward expansion mirrors that of other triggerfish species in the Canary Islands, including the first record in Madeira of ocean triggerfish in 2008 (Marrero et al., 2019; Wirtz et al., 2008), *C. maculata* in 2024 (Relvas & Wirtz, 2024) and *M. niger* in 2025 (Biscoito & Delgado, 2025).

In the short to medium term, the expected evolution of the ocean triggerfish population in the Canary Islands is expected to be similar to that observed for *B. capriscus* over the past four decades (Tejera, 2018), which transitioned from a rare species to a dominant component of demersal fisheries in some islands. The expansion of ocean triggerfish within the archipelago is already evident, with increasingly frequent sightings near La Palma, Gran Canaria and Lanzarote, as reported in official records from the government of the Canary Islands (Biota, 2024). Increasing water temperatures are expected to accelerate growth rates and promote the appearance of smaller individuals in shallow waters earlier than usual, possibly extending the observed period in shallow waters. Although there is fishing activity on this species in the southern part of El Hierro, its growth and expansion will not be negatively affected. The MPA ensures the reproduction of this species, acting as a source of colonisation towards other areas and islands (Bell, 2008).

In the long term, environmental conditions are predicted to change significantly in the coming decades (IPCC, 2022). Species with temperature‐dependent gonadal development, such as the ocean triggerfish, may experience a change in spawning phenology due to global ocean warming (Kutsyn, 2025; McQueen & Marshall, 2017). Drastic change in ocean temperature is expected to impact ocean triggerfish, as ecosystemic alterations (Bentley et al., 2021; Howell et al., 2021) could impair its reproductive output, food availability, survival rates and subsequent recruitment success (Graham & Harrod, 2009; Ottersen, 2000). Consequently, future research should investigate how ocean warming influences reproductive success, framed within three universal ecological responses: shifts in behaviour and phenology, poleward range expansion of tropical species and the reduction in body size typical of aquatic poikilotherms (Gardner et al., 2011; Kutsyn, 2025; Sheridan & Bickford, 2011).

## 5 CONCLUSION

Only mature or maturing *C. sufflamen* migrate to coastal habitats; in contrast, immature stages persist in oceanic and pelagic waters. The species exhibits no sexual dimorphism in total length or body weight. Within nesting sites, females engage in multiple spawning events due to asynchronous ovarian development. Reproductive behaviour is characterised by males constructing benthic nests and establishing territories defended through agonistic interactions. In addition, parental care of the eggs is present, along with sexual dichromatism, which manifests as dark body colouration – a visual signal of individual health and reproductive fitness. The distribution patterns of this species are closely linked to global warming, which is driving its expansion throughout the archipelago and towards higher latitudes. Evidence that mature individuals in the Canary Islands are larger than those in their natural range highlights the strong influence of local environmental factors, likely driven by the ecological constraints of being at the species' distribution limit.

## AUTHOR CONTRIBUTIONS

**Alberto Rodríguez‐Díaz:** data curation, formal analysis, investigation, writing – original draft, writing – review and editing. **Raül Triay‐Portella:** underwater and ROV images, data curation, formal analysis. **José A. González:** data curation, funding acquisition, project administration, sampling, resources. **José G. Pajuelo:** conceptualisation, formal analysis, investigation, supervision, coordination, writing – original draft, writing – review and editing.

## FUNDING INFORMATION

Financial support was received from the European Regional Development Fund (ERDF) in the framework of the Transnational Cooperation Programme MAC (Madeira‐Açores‐Canarias) projects MACAROFOOD (MAC/2.3d/015) and MARISCOMAC (MAC/2.3d/097).

## CONFLICT OF INTEREST STATEMENT

The authors of this article declare that they have no financial, professional or personal conflicts of interest that could have inappropriately influenced this work.

## Data Availability

The data that support the findings of this study are available from the corresponding author upon reasonable request.
